# Corpus callosal microstructure influences intermanual transfer in chimpanzees

**DOI:** 10.3389/fnsys.2013.00125

**Published:** 2013-12-31

**Authors:** Kimberley A. Phillips, Jennifer A. Schaeffer, William D. Hopkins

**Affiliations:** ^1^Department of Psychology, Trinity UniversitySan Antonio, TX, USA; ^2^Southwest National Primate Research Center, Texas Biomedical Research InstituteSan Antonio, TX, USA; ^3^Division of Cognitive and Developmental Neuroscience, Yerkes National Primate Research CenterAtlanta, GA, USA; ^4^Neuroscience Institute and Language Research Center, Georgia State UniversityAtlanta, GA, USA

**Keywords:** intermanual transfer, manual performance, fractional anisotropy, chimpanzees

## Abstract

Learning a new motor skill with one hand typically results in performance improvements in the alternate hand. The neural substrates involved with this skill acquisition are poorly understood. We combined behavioral testing and non-invasive brain imaging to study how the organization of the corpus callosum was related to intermanual transfer performance in chimpanzees. Fifty-three chimpanzees were tested for intermanual transfer of learning using a bent-wire task. Magnetic resonance and diffusion tensor images were collected from 39 of these subjects. The dominant hand showed greater performance benefits than the nondominant hand. Further, performance was associated with structural integrity of the motor and sensory regions of the CC. Subjects with better intermanual transfer of learning had lower fractional anisotropy values. The results are consistent with the callosal access model of motor programming.

## Introduction

Learning a new motor skill with one hand typically results in performance improvements in the alternate hand (Parlow and Kinsbourne, 1989; Grafton et al., 2002; Japikse et al., 2003). A typical paradigm is to have one hand learn and practice a specific motor task, and then to test whether the opposite (untrained) hand shows performance improvements. Inconsistent results have been reported as to whether greater performance improvements are shown from the dominant hand (DOM) to the nondominant hand (NDOM) (Milisen and Riper, 1939; Laszlo et al., 1970; Parlow and Kinsbourne, 1989; Halsband, 1992), or from the NDOM to the DOM (Hicks, 1974; Taylor and Heilman, 1980). The callosal access model postulates that motor programs are stored in the dominant hemisphere (in humans, typically the left), irrespective of the hand used during training. Thus, the DOM (in humans, typically the right) has direct access to these programs, whereas the nondominant (left) hand has indirect access via the corpus callosum (CC) (Taylor and Heilman, 1980). According to this model greater transfer of learning would be seen in DOM from NDOM training.

The CC, the major white matter tract connecting the two cerebral hemispheres, is crucial for interhemispheric transfer of information (Wahl and Ziemann, 2008). Two subdivisions of the CC appear to be most associated with intermanual transfer: that containing transcallosal fibers of the primary motor cortex (M1), and that containing transcallosal fibers of the supplementary motor area (SMA). Bonzano et al. (2011) reported a significant positive correlation between the structural integrity of the region containing M1 transcallosal fibers and an intermanual transfer task of reaction-time. In another study, reduced structural integrity of the anterior CC was associated with impairment in a coordinated bimanual task (Bonzano et al., 2008). The SMA is involved in the intermanual transfer of a newly acquired motor skill (Perez et al., 2007). In a test of learned sequential finger movements, greater activity was observed in the SMA when a skill transferred well than when the skill transferred poorly. Furthermore, blocking activity of the SMA through transcranial magnetic stimulation resulted in a blocking of intermanual transfer. Thus, the SMA is centrally involved in the interhemisphic transfer of motor skill learning, and we could reasonably expect the CC region where these transcallosal fibers cross to be associated with intermanual transfer performance.

Chimpanzees (*Pan troglodytes*) have evolved several motor characteristics in common with humans; including complex manipulation, use of feeding tools in the wild, corticospinal terminals in the ventral horn of the spinal cord, and the use of precision grips (Padberg et al., 2007). Thus, they are good models for understanding patterns of intermanual transfer and its association with the organization of the CC. Determining these relationships in chimpanzees will thus provide information about the fundamental aspects of neurobiological organization that underlie skilled motor actions and interhemispheric transfer.

The first aim of this study was to investigate intermanual transfer of learning in chimpanzees. Chimpanzees were presented with a skilled motor task that required them to use a specific hand to guide a metal washer off a curved rod. To determine which would result in greater performance improvements, some individuals trained on the DOM, others trained on the NDOM. Following the callosal access model, we hypothesized that those individuals trained on NDOM would see greater performance improvement. The second aim was to relate intermanual transfer to the structural integrity of the CC, specifically the regions containing transcallosal connections of M1 and SMA. We hypothesized that greater structural integrity of these regions would be associated with greater intermanual transfer.

## Methods

### Subjects

Fifty-three chimpanzees (*Pan troglodytes*) were tested on the behavioral component of the study (male = 18; female = 35), ranging in age from 9 to 47 years. Of these, 31 subjects trained on the DOM (male = 12; female = 19) and 22 subjects trained on NDOM (male = 6, female = 16). All of the chimpanzees resided at the Yerkes National Primate Research Center (Atlanta, Georgia). All aspects of this study were conducted in accordance with ethical guidelines associated with the care and use of nonhuman primates and with the approval of the Emory University Institutional Animal Care and Use Committee.

### Materials

**Figure 1:**
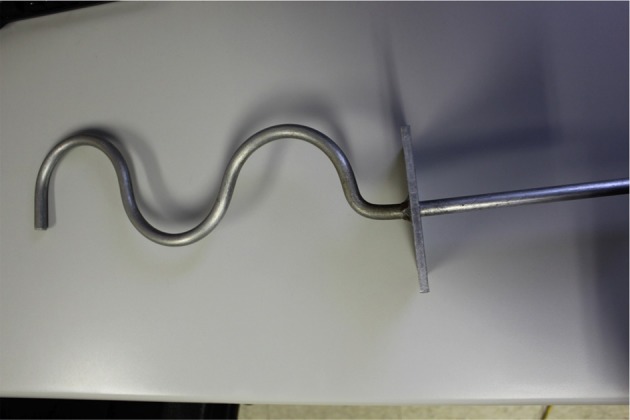
**Curved metal rod used to test intermanual transfer of learning**.

The device used for intermanual transfer testing was made using a 1 cm thick metal wire that has been curved into a pattern similar to an “S” shape but with an additional loop. The wire was welded to a metal plate measuring 11.35 by 11.35 cm which was designed to prevent the device from being pulled into the enclosure, and a handle was welded on the opposite side of the plate. The opening of each curve measured approximately 10 cm, and the distance from the wire end to the plate was 39 cm (see Figure 1).

### Procedure

#### Determination of DOM and NDOM hand

Individual's DOM and NDOM hands were determined based on hand preferences on a simple reaching paradigm (Hopkins et al., 2002). Briefly, hand preferences for simple reaching were measured by throwing small food items into the subject's outdoor enclosure and recording which hand was used to grasp the item. Hand use was recorded for 50 responses. Between each reaching response, the subject had to reposition or locomote at least 3 m before reaching for another food item. Based on the frequency in right and left hand use, a handedness index was computed following the formula [HI = (#R - #L)/(#R + #L)] where #R and #L represent the number of left and right hand responses. To simplify hand preference assignment in this paper, we classified subjects with positive HI scores as right-handed and subjects with negative HI scores as left-handed.

#### Intermanual transfer procedures

**Figure 2:**
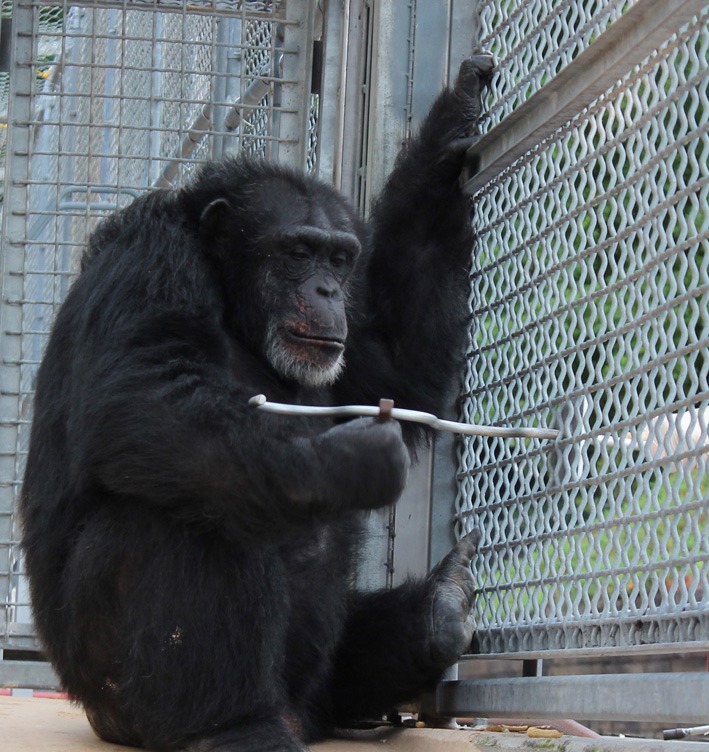
**Subject removing the nut from the curved metal rod with the target hand.** Note that the non-target hand is grasping the stationing stimulus.

All subjects were initially trained and subsequently tested on the S wire (see Figure 2) and reliably used only the designated hand before beginning testing on the intermanual transfer task. Each subject received 12 trials per test session and received 1 testing session per day.

At the start of each test session, the experimenter placed a stationing stimulus (a 9 cm PVC pipe extending into the subjects cage) to the left or right of the chimpanzee, depending on which hand they were going to use on the motor task during the session. During each trial, the chimpanzee had to grasp the stationing stimulus with the hand not being tested so as to prevent them from attempting to switch hands during the trial and thereby assure that they would use the designated hand during a session. A nut (2.5 cm in diameter) was then placed at the end of the bent wire and inserted into to the subject's home cage. The subject was then allowed to remove the nut from the wire with the target hand. If they were successful in removing the nut from the bent wire, they were rewarded with a small piece of food or squirt of juice.

If at any point during the trial the subject tried to use the non-target hand or mouth to remove the nut, the experimenter retracted the wire. If this occurred, the subject was not reinforced for this trial but the trial was repeated. During each trial, the experimenter recorded the time needed to remove the nut from the wire, and whether the subject was successful in removing the nut. Time was recorded from the moment the subject grasped the nut until it was successfully removed at the end of the wire. If the subject turned their attention away from the task or stopped working on the task, that attempt was not counted and the trial was repeated or if possible, the experimenter stopped the time until the subject re-engaged with the task. Each subject received 12 trials during each test session with the target hand. Test sessions were repeated until subject reached the performance criterion. Performance criterion was defined as each subject completing at least 10 of 12 trials within a single testing session in under 5 s. Once criterion was met, the subject was tested with the opposite hand following the methods and procedure described above.

#### Image acquisition and processing

We acquired non-invasive MRI and DTI scans from 39 chimpanzees who completed the intermanual testing (16 males, 23 females; average age = 20.19 years ± 7.34; 11 left- and 28 right-handers). As the animals needed to be anesthetized for this procedure, the collection of the brain images was coordinated with each subject's annual physical exam. Anesthesia was used only for the purpose of restraint and to keep the subject immobilized during their physical exam and collection of the brain images. Subjects were initially immobilized using ketamine (10 mg/kg) and subsequently anesthetized with propofol [40–60 mg (kg/h)] following standard procedures at the YNPRC. Subjects were under anesthesia for transport to and from the imaging facility, and remained anesthetized throughout the imaging procedure. Respiration rate, heart rate, and oxygen consumption were continually monitored by a veterinarian.

As described previously (Phillips and Hopkins, 2012), subjects were scanned on a Siemens 3.0 T Trio at the YNPRC. T1-weighted images were acquired using a 3D gradient echo sequence (pulse repetition = 2300 ms, echo time = 4.4 ms, number of signals averaged = 3, matrix size = 320 × 320, with 0.6 mm isotropic resolution). We acquired two sets of whole brain diffusion-weighted data with a single-shot EPI sequence with a *b*-value of 1000 s/mm^2^ with 64 diffusion directions; plus one image without diffusion weighting (*b*-value of 0 s/mm^2^). DTI data were acquired transaxially (FOV = 243 × 243) using 42 contiguous slices with no gap that covered the entire brain with resolution of 1.9 × 1.9 × 1.9 mm. Averages of two sets of diffusion-weighted data were collected per subject with phase-encoding directions of opposite polarity (left–right) to correct for susceptibility distortion. Acquisition time for both the MRI and DTI scans was approximately 1 h. After completing the DTI and MRI procedures the subjects were temporarily housed in a single cage for 6–12 h, to allow for the effects of anesthesia to wear off, after which they were returned to their home cage and social group.

#### Image quantification

**Figure 3:**
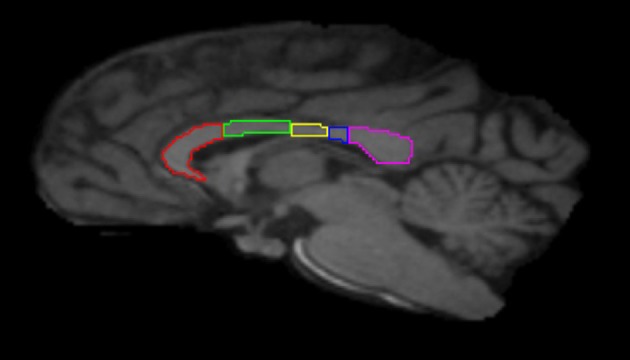
**Midsagittal section illustrating the subdivisions of the corpus callosum as determined by tractography.** Region I (red) = prefrontal cortex; Region II (green) = premotor and supplementary motor cortices; Region III (yellow) = motor cortex; Region IV (blue) = sensory cortex; Region V (violet) = parietal, temporal and occipital cortices.

**Figure 4:**
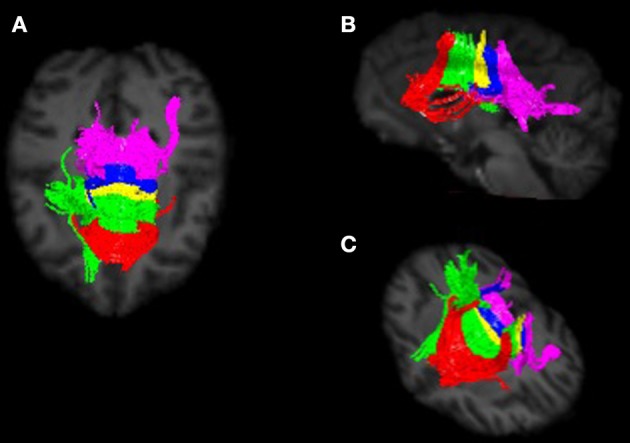
**Callosal fiber projections from a single male chimpanzee, displayed from (A) dorsal, **(B)** sagittal, and **(C)** oblique views.** Color distinguishes fibers projecting into cortical regions and are as follows: prefrontal (red), premotor and supplementary motor (green), motor (yellow), sensory (blue), and parietal, temporal and occipital (violet).

Image preprocessing steps included realignment, correction for head motion and eddy current distortion, and removal of non-brain tissue and were carried out with FSL tools (FMRIB Software Library; www.fmrib.ox.ac.uk/fsl). We used tractography to determine the fiber projections of the transcallosal fibers of M1 and SMA; this information was then used to subdivide the CC. Tractography was carried out using Analyze MR Diffusion Tensor Imaging based on fiber assignment by continuous tracking (FACT) algorithm (Jiang et al., 2006) with a fractional anisotropy (FA) threshold of 0.2 for initial seeding and stopping and a principal eigenvector angle stopping threshold of 60°. Landmarks used to define the projections into the cortex were the arcuate sulcus (for prefrontal cortex); the arcuate sulcus and central sulcus (for premotor, supplementary motor, and motor cortices); postcentral sulcus and parietooccipital sulcus (parietal cortex); lateral fissure (temporal cortex); and the inferior occipital sulcus and the parietooccipital sulcus (occipital cortex). The CC was then partitioned into five regions based upon fiber projections into specific cortical regions as follows: Region I, the most rostral region, into the prefrontal cortex; region II into premotor and supplementary motor cortices; region III into motor cortex; region IV into sensory cortex; and region V into parietal, temporal and occipital lobes (see Figures 3, 4). We were particularly interested in regions II and III in this investigation, as transcallosal connections of M1 are contained within Region III, and transcallosal connections of SMA and premotor cortex are contained within Region II (Hofer and Frahm, 2006; Phillips and Hopkins, 2012).

Measures of white matter integrity can be obtained using DTI, which quantifies the random diffusion of water molecules (Le Bihan, 1995). In white matter, water diffusion is anisotropic, with water diffusion greater along white matter fibers that are parallel rather than perpendicular to these fibers (Basser and Pierpaoli, 1996). Diffusion anisotropy measures the difference between these two directions of water diffusion. One of the more commonly reported measures of this diffusion is FA, the normalized standard deviation of the diffusivities (Basser and Pierpaoli, 1996). FA is influenced by anatomical features of white matter such as axon density, diameter, and myelination. To determine FA values for each callosal subdivision, each subject's MRI image was initially spatially registered to their respective DTI image using 3D voxel registration with a linear transformation using Analyze 10.0 (Analyze Direct, Overland Park, KS, USA). FA of each callosal region was measured in the midsagittal and two CC sections 1 mm lateral to the midsagittal using the above-defined callosal subdivisions to quantify the measure of diffusion anisotropy. Obtained values were then averaged for each subject for each callosal region. To reduce the effects of partial voluming, voxels at the edge were excluded.

## Results

**Figure 5:**
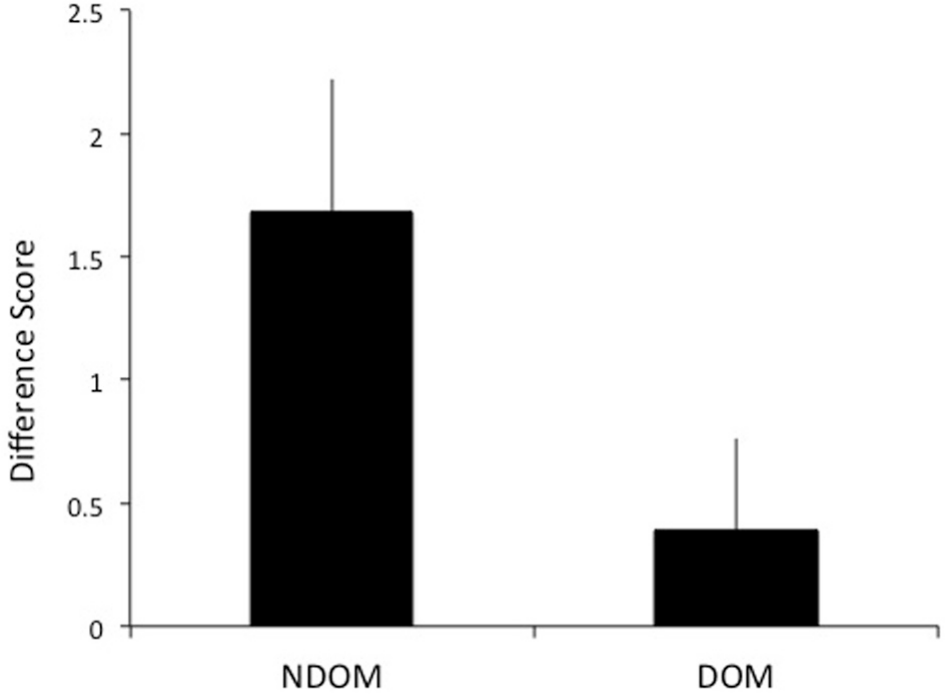
**Subjects who trained on the nondominant hand (NDOM) showed greater intermanual transfer of learning than those trained on the dominant hand (DOM), as indicated by higher difference scores**.

A difference score (DS) of the number of test sessions needed to reach criterion for the training and transfer hands was calculated to determine intermanual transfer performance. The DS reflects the savings in learning in the transfer hand. Larger DS scores reflect greater performance improvements. As hypothesized, greater transfer of learning was observed when switching to the DOM from NDOM training. Individuals who learned on the NDOM showed greater intermanual transfer of learning (as reflected by a higher DS score) than those who learned with DOM [*t*_(51)_ = 2.05, *p* = 0.023; mean NDOM = 1.68, *SE* = 0.54; mean DOM = 0.39, *SE* = 0.37; see Figure 5]. Whether the NDOM was right or left did not have an effect on intermanual transfer [*F*_(1, 48)_ = 0.009, *p* > 0.05].

To test the hypothesis that intermanual transfer performance is a function of structural integrity of the CC and age, a hierarchical multiple regression analysis was performed. Results of the analysis provided partial confirmation for the research hypotheses: negative associations were found between performance and structural integrity of Regions III and IV. Beta coefficients for the predictor FA of callosum region were: FA Region I, β = −0.170, *t* = −1.032, *p* = 0.309; FA Region II, β = −0.263, *t* = −1.633, *p* = 0.111; FA Region III, β = −0.326, *t* = −2.173, *p* = 0.036; FA Region IV, β = −0.403, *t* = −2.678, *p* = 0.011; FA Region V, β = 0.005, *t* = 0.022, *p* = 0.982. Addition of the variable age into the model did not significantly improve prediction for any callosal Region (*R*^2^ change Region 1 = 0.001. *F* = 0.0038, *p* = 0.954; *R*^2^ change Region 2 = 0.001. *F* = 0.017, *p* = 0.897; *R*^2^ change Region 3 = 0.078. *F* = 3.451, *p* = 0.071; *R*^2^ change Region 4 = 0.017. *F* = 0.776, *p* = 0.387; *R*^2^ change Region 5 = 0.004. *F* = 0.135, *p* = 0.716).

## Discussion

Our results show that in chimpanzees, the DOM showed greater performance benefits than the NDOM during intermanual transfer of a learned motor task. Interestingly, whether the NDOM was right or left did not influence the degree of intermanual transfer. It is possible that both left and right handed chimpanzees share a clear pattern of behavioral lateralization yet do not differ in terms of anatomical specialization with left hemisphere dominance for motor programs. In humans, while right-handedness is associated with left-hemisphere specialization, there is left hemisphere dominance for motor skills carried out with either hand (Serrien et al., 2006). Thus, in humans, both motor cortices are being guided by the left premotor cortex. According to the callosal access model (Taylor and Heilman, 1980) motor programs are stored in the left hemisphere, and greater transfer of learning would be seen in the DOM from NDOM training. Our results suggest that the callosal access model also applies to chimpanzees. Chimpanzees show task-specific population-level right handedness (Hopkins et al., 2007). Furthermore, unilateral grasping activates the contralateral motor-hand area, and white matter within the central precentral gyrus is correlated with performance differences in grasping skill (Hopkins et al., 2010).

The type of motor skill may be influential in terms of intermanual transfer performance. Stockel and Weigelt (2012) reported greater intermanual transfer for a spatial accuracy task in humans with training on the NDOM; whereas tasks requiring force or strength showed better intermanual performance when trained on the DOM. Kumar and Mandal (2005) investigated bilateral transfer of skill in humans using a mirror-drawing task, which requires spatial accuracy to successfully complete, and the same pattern of transfer of a motor program was demonstrated. Our results, using a task requiring spatial accuracy and fine motor skill, are in agreement with the hypothesis that intermanual transfer is influenced by the nature and complexity of the task.

Whether or not one learns a skill faster with the DOM is at least partly influenced by the type of skill; in humans, spatial accuracy tasks were learned better when practicing with the NDOM, while maximum forced production tasks were learned better when practicing with the DOM Stockel and Weigelt (2012). As we did not test both types of switches or different types of tasks in the present study we cannot exclude this possibility.

The second aim of this study was to relate microstructural integrity of the CC to intermanual transfer. We hypothesized that greater intermanual transfer would be associated with increased FA of the callosal regions containing transcallosal connections for cortical areas previously identified as involved in intermanual transfer, M1 and SMA. Instead, negative relationships between intermanual transfer performance and FA of callosal Regions III and IV were identified. Subjects who showed greater intermanual transfer of a learned motor task had lower FA of the callosal region containing fibers connecting motor and sensory regions. This would seem to indicate reduced structural integrity of the CC in those subjects who showed greater performance benefits. While no other relationships were significant, all showed a negative association between FA and DS score. While this may seem counterintuitive, studies relating callosal area, FA and function in humans are inconsistent. Anisotropy measures of the posterior callosal region correlated positively with pronounced left-hemisphere language lateralization (Westerhausen et al., 2006). Westerhausen et al. suggested this indicated individuals showing extreme left lateralization of language had a greater number of axons and/or thicker myelin sheaths in posterior callosal regions than individuals who showed less language lateralization. In individuals with autism, FA correlated positively with performance IQ in the total CC and subregions (Alexander et al., 2007). Sisti et al. (2012), using a similar parcellation of the CC in humans based on tractography, reported FA in callosal regions connecting prefrontal cortices predicted performance on the acquisition of a novel bimanual coordination task.

In a study most similar to the present study in terms of methodologies (Bonzano et al., 2008), a positive correlation was found between FA of the anterior CC and performance of a bimanual task.

Another consideration concerns age differences in CC macro- and micro-structure that could influence intermanual transfer. Structural MRI has demonstrated callosal size changes during development in chimpanzees (Hopkins and Phillips, 2010) and humans (Allen et al., 1991; Pujol et al., 1993; Hasan et al., 2008). FA changes are related to these developmental patterns of callosal size in humans (Lebel et al., 2012; Yap et al., 2013). These structural changes of the CC across development influence behavior. For example, less lateralized task processing occurs in cognitive and motor tasks, and is associated with reduced callosal area in older adults (Muller-Oehring et al., 2007; Langan et al., 2010) While age was not significant to intermanual performance in the present study, other variables should be explored in lieu of or combination with FA to improve the predictability of intermanual transfer.

The microstructure of the transcallosal fibers of primary motor cortices reflects the capacity for interhemispheric inhibition (Wahl et al., 2007; Koerte et al., 2009). Wahl et al. reported a positive relationship between microstructural integrity (FA) and strength of inhibition in adults. Koerte et al. found a similar relationship across development (7–26 years), but Fling et al. (2013) suggest these data were largely driven by the child group. Further analysis indicated that the observed relationship between FA and strength of inhibition may actually show an opposite pattern in adults. Thus, adults with higher microstructural integrity may have reduced interhemispheric inhibition during volitional cortical activity. In support of this hypothesis, Fling et al. report higher CC microstructural integrity was associated with poorer performance on bimanual tasks requiring a large degree of interhemispheric inhibition.

FA is undoubtedly influenced by multiple characteristics of water diffusion, including myelination, axon diameter and axon density. Hopkins et al. (2012) examined axon density across regions of the CC in chimpanzees. The highest fiber densities (for both small and large myelinated axons) were reported in the genu and splenium (the rostrum was not analyzed in this study), and there was no difference between genu and splenium in axon density. No differences were found across the CC for density of large axonal fibers. Considering small axonal fibers, the highest density was in the genu. If FA primarily reflects axonal density rather than axon diameter or myelination, as has been posited by some [e.g., Wahl et al. (2007)], then FA of these callosal regions in chimpanzees should show a similar pattern to the axon density pattern reported by Hopkins et al. However, it does not. Phillips and Hopkins (2012), in an examination of FA in chimpanzee callosal subdivisions, reported the posterior region of the CC (which would include the splenium as discussed above) had the highest FA value, and was significantly higher than the genu. Thus, FA and fiber density data do not precisely match.

In sum, chimpanzees showed greater performance benefits when switching to the DOM from NDOM during intermanual transfer of a learned motor task, and intermanual transfer performance was associated with variation in white matter integrity of regions of the body of the CC that contain motor and sensory projections. We suggest that in chimpanzees, lower structural integrity in these regions of the CC indicates less interhemispheric inhibition, which leads to greater intermanual transfer of learning.

## Author contributions

Kimberley A. Phillips and William D. Hopkins designed the study. William D. Hopkins and Jennifer A. Schaeffer collected the data; Kimberley A. Phillips analyzed the data. Kimberley A. Phillips and William D. Hopkins wrote the manuscript.

### Conflict of interest statement

The authors declare that the research was conducted in the absence of any commercial or financial relationships that could be construed as a potential conflict of interest.
